# First-Principles Understanding of Mono- and Dual-Emissions in AZnOS:Bi^3+^ (A = Ba, Ca) Phosphors

**DOI:** 10.3390/ma18030657

**Published:** 2025-02-02

**Authors:** Quanzhi Kougong, Bibo Lou, Mikhail G. Brik

**Affiliations:** 1 College of Electronic and Optical Engineering & College of Flexible Electronics (Future Technology), Nanjing University of Posts & Telecommunications, Nanjing 210023, China; 1322028818@njupt.edu.cn; 2 School of Optoelectronic Engineering, Chongqing University of Posts and Telecommunications, Chongqing 400065, China; 3 Centre of Excellence for Photoconversion, Vinča Institute of Nuclear Sciences—National Institute of the Republic of Serbia, University of Belgrade, 11000 Belgrade, Serbia; 4 Institute of Physics, University of Tartu, W. Ostwald Str. 1, 50411 Tartu, Estonia; 5 Faculty of Science and Technology, Jan Długosz University, Armii Krajowej 13/15, PL-42200 Częstochowa, Poland; 6 Academy of Romanian Scientists, Ilfov Str. No. 3, 050044 Bucharest, Romania

**Keywords:** bismuth, dual-emission, luminescence mechanism, first-principles calculations

## Abstract

The AZnOS:Bi^3+^ (A = Ba, Ca) phosphors exhibit mono- and dual-emission phenomena based on the different choices of cation, making them an ideal prototype for dual-emission mechanism studies of Bi^3+^ ions. Here, first-principles calculations were performed to investigate the site occupancy, defect levels, and luminescence properties of the AZnOS:Bi^3+^ systems. The formation energy calculations show that the bismuth dopants are mainly in the trivalent charge state, with the Bi^3+^ ions preferring the Ca sites in CaZnOS but the Zn sites in BaZnOS. Such cation-selective occupancy mainly results in the difference between the mono- and dual-emission phenomena in the two hosts. The excitation and emission energies predicted by calculations are in good agreement with the measurements. Our calculations show that the lowest excited state ^3^P_0,1_ provides the dominant emission in both CaZnOS:Bi^3+^ and BaZnOS:Bi^3+^ phosphors. In light of the experimental and theoretical results, the metastable excited state of Bi^2+^ + h_VBM_ (hole at the valence band maximum) is the origin of the additional emission bands in BaZnOS:Bi^3+^. These results provide the basis of emission band tuning and novel material design for Bi^3+^-doped phosphors.

## 1. Introduction

In contrast to the general mono-emission phenomenon, some single Bi^3+^-doped phosphors exhibit dual-emission bands, e.g., Y_2_O_3_:Bi^3+^ [1], MgGa_2_O_4_:Bi^3+^ [2]. They provide an extra red-shifted emission band in addition to the commonly observed green or blue emission band. Recently, such dual-emission character has become attractive due to the potential application in anti-counterfeiting [3], temperature sensing [4], and healthcare lighting [5], where Bi^3+^ ions, as an important activator, can help to control the trapping and detrapping processes through valence or conduction band engineering, endowing the host matrix with multifunctional phosphorescent performances. Bi^3+^-activated dual-emission materials with a wide spectral response range can effectively promote the effective dynamic anti-counterfeiting properties. Understanding the dual-emission mechanisms of Bi^3+^ dopants is crucially important to improve the luminescence performance and expand the application boundaries of the Bi^3+^-doped phosphors. On the one hand, the spectral assignment and the origin of the emission bands should be clarified. On the other hand, the schemes for tuning mono-emitting phosphors into dual-emitting ones should be explored. However, the complex optical transitions of Bi^3+^-doped phosphors (except for the ^1^S_0_ → ^3^P_0,1_ transition between two electron configurations of a Bi^3+^ ion, in which there are excitation and emission processes involving charge transfer between a Bi^3+^ ion and the host, such as the valence band to Bi^3+^ charge transfer, usually denoted as CT, and the ionization of an electron from Bi^3+^ to the condition band, usually denoted as metal–metal charge transfer (MMCT)) always bring challenges in the spectral assignment and the luminescence studies. Several potential dual-emission mechanisms have been proposed, including the site-selective occupancy of dopants [6], influence of bismuth ions with lower valence states [7], formation of Bi pairs or clusters [8], and Jahn–Teller distortion of the local environment [9]. It is difficult to obtain an explicit understanding of dual-emission mechanisms using experimental methods alone.

The AZnOS (A = Ca, Ba) crystals are promising hosts that exhibit excellent piezoelectric and luminescent properties, and the different cations at the A site are responsible for the mono- and dual-emission phenomena. Under 366 nm excitation, Bi-doped CaZnOS and BaZnOS show strong broad emission bands with their peaks at about 485 and 507 nm, respectively, but an extra broad emission band with the peak at 627 nm was only observed in BaZnOS:Bi under 297 nm excitation [10]. The experimental studies indicated that the A sites are the most suitable and stable sites for the Bi ions to occupy according to the Rietveld refinement, and the dopant Bi ions were found to be in the trivalent state in CaZnOS. For the Bi dopants in BaZnOS, the divalent and trivalent charge states coexist. The dual-emission was attributed to bismuth dopants with different charge states. However, such an explanation faced inconsistencies with the excitation spectra and the decay time properties, e.g., the decay time of 627 nm emission (884 ns) in BaZnOS:Bi^3+^ is close to the emission peak at 485 nm (773 ns) in CaZnOS:Bi^3+^ [10]. And similar dual-emissions were observed in Sb^3+^-doped CaZnOS [11]. In addition to the luminescence properties, the piezoelectric properties of Bi^3+^-doped AZnOS have also attracted great interest. It exhibits intense mechanoluminescence in the visible light region with potential applications in pressure sensing [12,13] and stress-based anti-counterfeiting technologies [14]. A systematic investigation of the electronic structures and luminescence mechanisms of Bi^3+^-doped AZnOS phosphors will provide insights into potential strategies for enhancing performance, as well as the design of novel phosphors with exceptional piezoelectric and luminescent properties. First-principles calculations have been widely used as a powerful tool to investigate and elucidate the excitation, relaxation, and emission processes of a large number of bismuth-doped phosphors [15]. Previous theoretical studies on AZnOS:Bi^3+^ (A = Ca, Ba) systems focused on the ground-state properties of hosts and dopants, e.g., Zhang et al. studied the elastic and tensile properties [16] and Pan et al. studied the site occupancy of bismuth by the binding energy comparison [10]. However, these studies lack the excited-state properties that are essential for the investigation of the dynamic emission processes.

Herein, first-principles calculations were employed to study the luminescence properties of bismuth in the oxysulfide AZnOS (A = Ca, Ba), in particular, the dual-emission mechanism of BaZnOS:Bi^3+^. The local environments of the dopants and the formation energies of the defects in AZnOS:Bi^3+^ systems were studied to clarify the site occupancy and the valence state of the bismuth dopants. The defect levels and the electronic structures were studied to obtain the optical luminescence properties of the Bi^3+^ ions in the two hosts and to reveal the origin of the dual-emission bands. The emission properties of the lower-charge-state bismuth ions (Bi^2+^ and Bi^+^) were also discussed in order to clarify the hypothesis. Our studies on the mono- and dual-emission mechanisms of AZnOS:Bi^3+^ systems can assist in the novel material design and the emission adjustment, thereby expanding the potential applications of Bi^3+^-doped phosphors, such as achieving high-quality white light by controlling the relative intensities of the mono- and dual-emission peaks.

## 2. Materials and Methods

In our calculation, the geometric structure relaxations were performed in the Strongly Constrained and Appropriately Normed (SCAN) functional [17] within the VASP code [18]. Semicore electrons were explicitly treated with the recommended projector augment wave pseudopotentials [19]. Spin–orbit coupling (SOC) was included for bismuth- and antimony-containing supercells. The cutoff energy of the plane-wave basis was set to 520 eV, while the convergence criteria were $10^{-5}$ eV for electronic energy minimization and 0.02 eV/Å for Hellman–Feynman forces on each atom. Based on the relaxed equilibrium geometric structures, the PBE0 functional [20] with 25% Hartree–Fock exchange was used in obtaining better electronic structure and photoluminescence properties. The defects containing BaZnOS crystals were modeled using a 3 × 1 × 2 supercell, while a supercell with new base vectors of 3(a+b), 2(a−b), and c was utilized for CaZnOS. The 7×7×5 (CaZnOS) and 7×3×5 (BaZnOS) *k*-point grids were used in sampling the Brillouin zone of the unit cell, while only one *k* point *Γ* was used for all the supercells.

The formation energy of a defect *X* in the charge state of *q* can be derived as follows [21]:

(1) $$E^{f}(X^{q},E_{F})=E_{tot}\left[X^{q}\right]-E_{tot}\left[bulk\right]-\sum_{i}n_{i}\mu_{i}+qE_{F},$$

where $E_{tot}$ is the total energy of the optimized supercells, $n_{i}$ are the numbers of atoms in elements *i* that are added to ($n_{i}$ > 0) and/or removed from ($n_{i}$ < 0) the perfect supercell, and $\mu_{i}$ are the corresponding chemical potentials of these species. The Fermi energy level $E_{F}$ represents the chemical potential of the electrons in the host. The thermodynamic charge transition level $\epsilon (q_{1}/q_{2})$ was utilized to predict the positions of defect levels, and it is defined as the Fermi level where the defect formation energies of $X^{q_{1}}$ and $X^{q_{2}}$ are equal. It can be derived from

(2) $$\epsilon (q_{1}/q_{2})=\frac{E^{f}\left(X^{q_{1}},\;{\;E}_{f}=0\right)-E^{f}\left(X^{q_{2}},\;{\;E}_{f}=0\right)}{q_{2}-q_{1}},$$

where post hoc corrections to the total energy of charged defects are applied according to the method proposed in Ref. [22].

For Bi^3+^ ion-doped systems, the dominant emission can originate from the equilibrium structure of the 6s^1^6p^1^ (^3^P_0,1_) excited state and the charge transfer state, including the MMCT excited state (Bi^4+^ + *e*) and the CT excited state (Bi^2+^ + *h*). Since an extra extended electron or hole in the energy band of the host hardly influences the ligand environment and luminescent property of the emission center, the equilibrium geometric structures for MMCT and CT excited states were approximately obtained by geometry optimization for the supercell with electron number setting corresponding to Bi^4+^ and Bi^2+^ [23,24]. The ^3^P_0,1_ excited state was approximated by constraining the electron occupancy to (6s_1/2_)^1^(6p_1/2_)^1^ for $AZnOS:Bi^{3+}$, where the 6s_1/2_ and 6p_1/2_ are Kohn–Sham orbitals obtained with the SCAN + SOC functional. For the optical transition energy calculations, the MMCT excitation energy is approximated by the difference in the energy of the $AZnOS:{Bi}^{4+}$ system plus an electron in CBM from that of the $AZnOS:{Bi}^{3+}$ system. Similarly, the CT absorption and emission energies can be calculated as the difference in the energy of $AZnOS:Bi^{2+}$ plus a hole from that of the $AZnOS:Bi^{3+}$ system. For optical transitions between ^1^S_0_ and ^3^P_0,1_, the standard ΔSCF-DFT procedure was employed.

## 3. Results and Discussion

### 3.1. Geometric and Electronic Structures of Hosts

**Figure 1:**
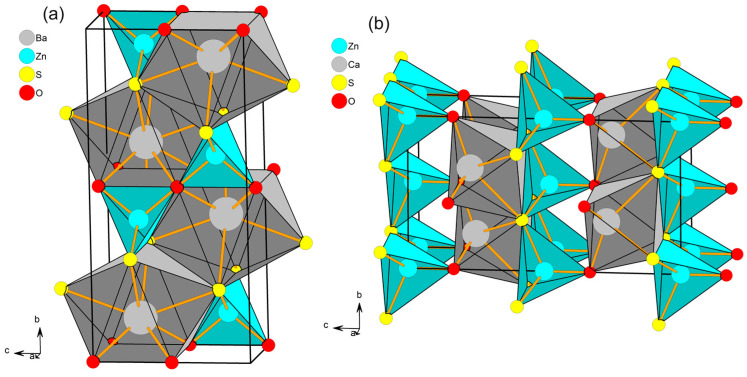
The crystal structure of the BaZnOS (**a**) and CaZnOS (**b**) hosts.

As shown in Figure 1, the crystal structures of CaZnOS and BaZnOS compounds belong to the hexagonal (P63mc, No. 186) and orthorhombic (Cmcm, No. 63) structures, respectively.

The difference in ionic radii between Ca^2+^ (1.00 Å, coordination number CN = 6) and Ba^2+^ (1.35 Å, CN = 6; 1.42 Å, CN = 8) ions has great influence on the local structure of the two hosts. For CaZnOS, all the Ca cations are located at the Wyck 2b sites are surrounded by three oxygen ligands (bond length BL = 2.27 Å) and three sulfur ligands (BL = 3.05 Å). The Zn cations located at the Wyck 2a sites are surrounded by one oxygen ligand (BL = 1.90 Å) and three sulfur ligands (BL = 2.35 Å). However, there are eight ligands for Ba sites located at the Wyck 4c sites in BaZnOS, including four oxygen ligand and four sulfur ligands with bond lengths of 2.86 Å and 3.26 Å, respectively. For the Zn cation in BaZnOS, it is also located at the Wyck 4c sites and is surrounded by two oxygen anions (BL = 1.98 Å) and two sulfur anions (BL = 2.32 Å). Traditionally, the site occupancy of dopants is judged by the ionic radius. Here, the doped Bi^3+^ ions (1.17 Å, CN = 8; 1.03 Å, CN = 6) more likely occupy the Ca^2+^ cation sites (1.00 Å, CN = 6) rather than the Zn^2+^ sites (r = 0.60 Å, CN = 4) for CaZnOS. However, the occupancy of the Bi^3+^ dopant in BaZnOS remains confusing because the ionic radius of Bi^3+^ is remarkably mismatched with those of the two cation sites. Previous experimental work has pointed out the difficulty in distinguishing the exact site occupancy of Bi from the XRD patterns [10] and proposed the requirements of the first-principles calculations.

**Figure 2:**
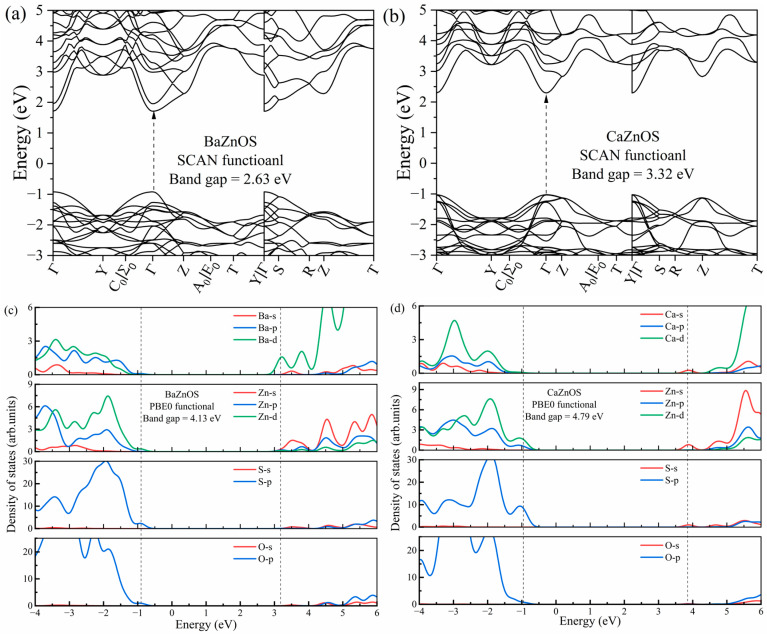
The band structure calculated with SCAN for (**a**) BaZnOS and (**b**) CaZnOS hosts, and the PBE0-calculated total and partial density of states (DOSs) for (**c**) BaZnOS and (**d**) CaZnOS hosts.

Figure 2 shows the SCAN-calculated band structures of the pristine AZnOS (A = Ca, Ba), where the valence band maximum (VBM) and conduction band minimum (CBM) both locate on the *Γ* point, implying that both materials are direct gap semiconductors. The Kohn–Sham band gaps calculated with the SCAN functional are 3.32 eV and 2.63 eV for A = Ca and Ba, respectively. The results are better than the values obtained with the Perdew–Burke–Ernzerhof functional (PBE) [25], e.g., 2.81 eV for CaZnOS and 2.27 eV for BaZnOS, while they remain seriously underestimated compared to the experimentally reported optical band gaps. The band gap values are 4.50 eV for CaZnOS and 4.00 eV for BaZnOS, characterized by host absorption spectra and diffuse reflectance spectroscopy [26]. For Bi^3+^ dopants, the spectral assignments and mechanism studies of the charge transfer transitions involving the band edge orbitals will be strongly influenced by the accuracy of the band gap values. Thus, PBE0 calculations were utilized to improve the description of the band gap value, which provides band gap energies (Eg) of 4.79 eV and 4.13 eV for CaZnOS and BaZnOS, respectively. The PBE0-calculated density of states (Figure 2c,d) shows that the tops of the valence bands for both two AZnOS are both dominated by the S-p orbitals, and partially contributed by the O-p, Zn-d orbitals. The O-p orbitals become important from −0.5 eV below the valence band. The conduction band of both hosts is the mixture of four elements for CaZnOS, including the Ca-s, Zn-s, S-s, and O-s orbitals, while it is dominated by the Ba-d orbitals for BaZnOS.

### 3.2. Formation of the Intrinsic Defects and Bi Dopants

**Figure 3:**
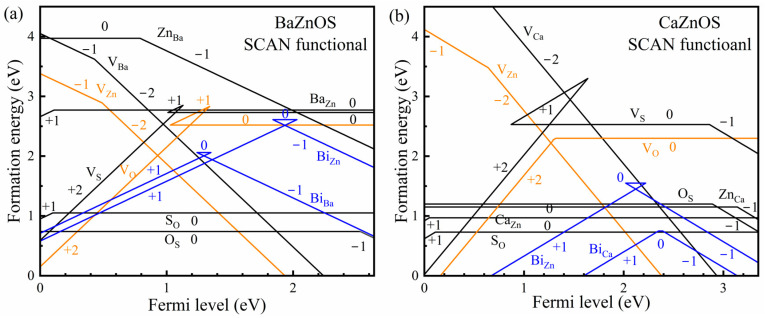
The formation energy diagrams of the intrinsic defects and the dopants of Bi ions substituting the A site or the Zn site in (**a**) BaZnOS:Bi^3+^ and (**b**) CaZnOS:Bi^3+^ materials.

Figure 3 shows the formation energies of the intrinsic defects, including *A* site or Zn cation vacancies (*V*_Ca_, *V*_Ba_ or *V*_Zn_), oxygen vacancies (*V*_O_), sulfur vacancies (*V*_S_), cation antisite defects (Ca_Zn_, Zn_Ca_, Ba_Zn_ and Zn_Ba_), anion antisite defects (O_S_, S_O_), as well as those of Bi substituting the *A* sites or Zn sites (Bi_Ca_, Bi_Ba_ or Bi_Zn_). In Figure 3, the employed chemical potentials of the components are determined by considering the following conditions. In the formation of *A*ZnOS compounds from raw materials (*A*S and ZnO bulks), there is an enthalpy change $\Delta H=\mu_{AZnOS(bulk)}-\mu_{AS\left(bulk\right)}-\mu_{ZnO\left(bulk\right)}$. Since the equation $\mu_{AZnOS(bulk)}=\mu_{A}+\mu_{Zn}+\mu_{O}+\mu_{S}$ is linked with the stable AZnOS compounds, the ΔH can be equally separated in approximation, giving the two following equations:

(3) $$\mu_{A}+\mu_{S}=\mu_{AS\left(bulk\right)}+\Delta H/2$$

(4) $$\mu_{Zn}+\mu_{O}=\mu_{ZnO\left(bulk\right)}+\Delta H/2.$$

Avoiding confusion from the choice of reference, the following equations are introduced: $\mu_{A}=\mu_{A\left[bulk\right]}+\Delta \mu_{A}$, $\mu_{Zn}=\mu_{Zn\left[bulk\right]}+\Delta \mu_{Zn}$, $\mu_{O}=1/2\mu_{O_{2}\left[gas\right]}+\Delta \mu_{O}$, $\mu_{S}=\mu_{S\left[bulk\right]}+\Delta \mu_{S}$, which can be used to rewrite Equations (1) and (2). Meanwhile, we take the condition of $\Delta \mu_{A}=\Delta \mu_{S}$ and $\Delta \mu_{Zn}=\Delta \mu_{O}$ for reference, and ignore the overall shift of chemical potential ΔH/4 for all the elements, where the values of ΔH are calculated to be −0.03 eV and −0.87 eV for A = Ca and Ba, respectively. The referenced element chemical potentials are as follows:

(5) $$\Delta \mu_{A}=\Delta \mu_{S}=1/2(\mu_{AS\left[bulk\right]}-\mu_{A\left[bulk\right]}-\mu_{S\left[bulk\right]})$$

(6) $$\Delta \mu_{Zn}=\Delta \mu_{O}=1/2(\mu_{ZnO\left[bulk\right]}-\mu_{Zn\left[bulk\right]}-1/2\mu_{O_{2}\left[gas\right]}).$$

The defect concentration is related to the Gibbs energy of formation. For solid phases, it is always approximated by the formation energy, expressed as follows: $c/N_{sites}=\omega exp(-E^{f}/k_{B}T)$, where the defect concentration c is strongly influenced by the parameter $E^{f}$ (formation energy) rather than by T (the temperature at which the defects reach thermal equilibrium distribution), $N_{sites}$ (the numbers of available atomic sites), or ω (the degeneracy factor of defects). For the two *A*ZnOS (*A* = Ba, Ca) hosts, the Fermi levels are dominated by the charge balance between the cation and anion vacancies located close to the valance band, as shown by the orange line of $V_{Zn}^{2-}$ and $V_{O}^{2+}$ in Figure 3. The formation energies of the antisite defects (O_S_ and S_O_) show lower formation energies at around 1.0 eV in the two *A*ZnOS, acting as the dominant intrinsic defects. However, the formation of O_S_ and S_O_ defects hardly influences the Fermi level of materials, as they are always in neutral charge states. The antisite defects between the two cation sites show quite different properties for the two *A*ZnOS. The formation energies of the Ca_Zn_ and Zn_Ca_ antisite defects in CaZnOS are about 1.0 eV, remarkably lower than the Ba_Zn_ and Zn_Ba_ defects in BaZnOS. This is consistent with the large ionic radii differences between the Ba^2+^ ions and Zn^2+^ ions. The *V*_O_ and V_S_ defects are mainly in “+2” charge states for the two *A*ZnOS, and the formation energies of *V*_O_ are slightly lower than those of V_S_. The V_Ca_, V_Ba_, and V_Zn_ defects are in “−2” charge states, where the V_Zn_ defects show the lowest formation energy.

With the doping of bismuth ions, e.g., increasing the amount of Bi_2_O_3_ during preparation, the Fermi level will increase and the charge balance case will shift from $V_{Zn}^{2-}$ and $V_{O}^{2+}$ to $V_{Zn}^{2-}$ and ${Bi}_{Ca}^{+}$ in CaZnOS:Bi^3+^ materials. The doped bismuth ions are mainly “+3” charge states in CaZnOS:Bi^3+^, and it is easier for Bi^3+^ ions to substitute the Ca^2+^ site than the Zn^2+^ site. The concentration of $V_{Zn}^{2-}$ defects will remarkably increase, for charge balance, the ${Bi}_{Ca}^{+}$ defects. In BaZnOS:Bi^3+^ materials, the doped bismuth ions are also in “+3” charge states, no matter whether Bi ions substitute the Ba^2+^ or Zn^2+^ sites. The formation energies of ${Bi}_{Ba}^{+}$ and ${Bi}_{Zn}^{+}$ defects are similar, and the Bi^3+^ dopants slightly prefer occupying the Zn^2+^ cation sites. Meanwhile, the formation energies of ${Bi}_{Ba}^{+}$ and ${Bi}_{Zn}^{+}$ defects are close to the $V_{O}^{2+}$ defects in BaZnOS:Bi^3+^; the Fermi level position and the intrinsic defect concentration in BaZnOS:Bi^3+^ are hardly influenced by the Bi^3+^ doping amount adjustment. In short, the site occupancies and the charge states of the dopants can be determined well by the formation energy calculations, which are greatly consistent with those from the empirical model [10]. And the charge state of the Bi ions in the Ba site of BaZnOS is also in the trivalent rather than the divalent state.

### 3.3. Electronic Properties of Bi^3+^ Dopants

**Figure 4:**
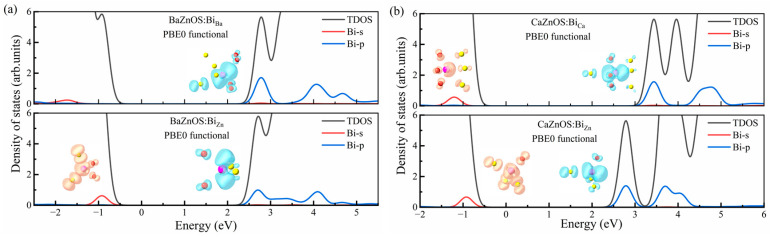
The PBE0 + SOC-calculated partial DOSs of Bi^3+^ 6s and 6p orbitals for the ground state of (**a**) BaZnOS:Bi^3+^ and (**b**) CaZnOS:Bi^3+^ materials, and the charge density profiles of the Bi^3+^ 6s and 6p orbitals after the localization of a hole and an electron, respectively.

Figure 4 shows the partial DOSs of 6s and 6p orbitals in Bi^3+^ in the ground states of *A*ZnOS:Bi^3+^ (*A* = Ca, Ba), which were calculated with the PBE0 functional that included SOC. In the two hosts, the 6p orbitals of Bi^3+^ dopants are always near the CBM, no matter whether Bi^3+^ substitutes *A* sites or Zn sites. The 6s orbitals of Bi^3+^ dopants are near the VBM in the Zn sites, while the 6s orbitals are deep in the valence band for Bi^3+^ when substituting the *A* sites. The Bi^3+^-6s orbital together with the ligand O-2p and S-3p orbital form an anti-bonding orbital, whose energy position is determined by the nephelauxetic effect between the Bi^3+^ ion and the ligands. The Bi^3+^-6s orbital contains substantial contributions from the *p* orbitals of the nearest three oxygen ligands and three sulfur ligands. Thus, the S-3p orbitals, which dominate the top of the valence band, contribute more to the localized Bi^3+^-6*s* state than the O-2p orbitals. In Figure 4, the charge density profiles of the hole and electron localized on the Bi^3+^-6s and Bi^3+^-6p orbitals, respectively, are also plotted in order to exhibit such picture more intuitive. Compared to the dispersed Bi^3+^-6s orbitals, the Bi^3+^-6p orbital was mainly distributed along the one axis. For the Bi^3+^ dopants substituting the Ca sites in CaZnOS:Bi^3+^, the next-nearest sulfur with a bond length of ~3.5 Å has also contributed to the Bi^3+^-6s orbitals. This clearly shows that the geometric structure relaxation properties of Bi dopants in different charge states are correlated with the electronic structure of the Bi 6s and 6p orbitals.

**Figure 5:**
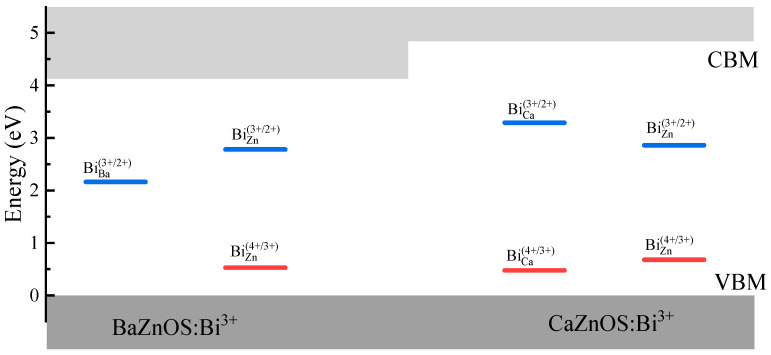
The thermodynamic charge transition level of Bi^3+^ dopants in *A*ZnOS (*A* = Ba, Ca) hosts, where the valence band maximum was set as reference.

In our previous work [24], it was pointed out that the luminescence processes of Bi^3+^ dopants can be predicted by their defect levels in the band gaps, e.g., (1) the ^3^P_0,1_ emission will dominate when the Bi^3+^ ions act as both stable electron and electron traps; (2) the CT emission will dominate when the Bi^3+^ ions act as stable electron traps only; (3) the MMCT emission will dominate when the Bi^3+^ ions act as stable hole traps only. As shown in Figure 5, thermodynamic charge transition levels calculated by the PBE0 functional are used to describe the trap depths of Bi^3+^ ions acting as hole or electron traps. In BaZnOS:Bi^3+^, the (4+/3+) transition levels of Bi ions in the *A* sites cannot be found in the band gap, and the doped Bi^3+^ ions only act as deep electron traps. Thus, the CT (Bi^2+^ + *h*_VBM_) excited state is predicted to be the lowest excited state and dominates the emission for Bi^3+^ ions in the *A* sites, which is calculated to be 1.54 eV. For Bi^3+^ substituting the Zn sites of BaZnOS:Bi^3+^, the (4+/3+) and (3+/2+) transition levels are both in the band gap. Thus, the lowest Bi^3+^ excited state should be ^3^P_0,1_, which can give an emission energy of 1.74 eV. For CaZnOS:Bi^3+^, regardless of whether Bi^3+^ substitutes the *A* sites or the Zn sites, (4+/3+) and (3+/2+) transition levels are always in the band gap, and the ^3^P_0,1_ excited states will always dominate the emission. The ^3^P_0,1_ emission energies of Bi^3+^ dopants are calculated to be 2.57 eV in *A* sites and 1.56 eV in Zn sites.

Considering the above site occupancy studies, we confirm that the experimentally measured strong broad emission band, peaking at about 485 nm (2.55 eV) in CaZnOS:Bi^3+^, originates from the ^3^P_0,1_ excited state of Bi^3+^ in the Ca site. The dominant broad emission band, peaking at about 627nm (1.97 eV) in BaZnOS:Bi^3+^, originated from the ^3^P_0,1_ excited state of Bi^3+^ in the Zn site. Although we improved the assignments of the dominant emission in *A*ZnOS:Bi^3+^ (*A* = Ca, Ba), the additional emission peaking at about 507 nm (2.44 eV) in BaZnOS remains unknown and requires further studies of the detailed luminescence mechanism.

### 3.4. Excitation and Luminescence of Bi^3+^ Dopants

**Figure 6:**
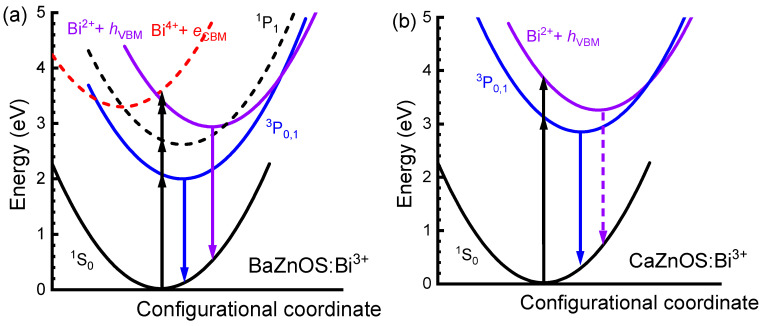
The configuration coordinate diagrams of the potential surfaces of Bi^3+^ as a function of the generalized configuration coordinate for (**a**) BaZnOS:Bi^3+^ and (**b**) CaZnOS:Bi^3+^ materials, where ^1^S_0_, ^3^P_0,1_, and ^1^P_1_ denote the ground, the lowest triplet, and the singlet 6s6p excited states of Bi^3+^, respectively. Bi^4+^ + *e*_CBM_ simulates Bi^4+^ with one electron at CBM, representing the lowest MMCT excited state, and Bi^2+^ + *h*_VBM_ simulates Bi^2+^ with a loose hole at VBM, representing the lowest CT excited state.

Based on the optimized equilibrium structures for both ground and various Bi^3+^-related excited states for the two hosts, we obtain the excitation and emission energies for various transitions, as listed in Table 1. To illustrate the luminescence processes, a schematic configuration coordinate diagram is constructed based on these results, as shown in Figure 6. For Bi^3+^ substituting the Zn site in BaZnOS, the A band, CT, and MMCT excitation energies are 2.29, 3.60, and 3.67 eV, respectively, according to our calculations. Using the 6s6p excited state splitting in PBE0 + SOC calculations [27], we estimated the ^1^S_0_ → ^1^P_1_ excitation to be 2.91 eV. Since the ^3^P_0,1_ is the lowest excited state, all the above excitations together with the host related absorption, approximated by the band gap 4.13 eV, were able to provide ^3^P_0,1_ emission. This is consistent with the various excitation bands in the experiment peaking at about 450 nm (2.75 eV), 366 nm (3.38 eV), and 297 nm (4.17 eV). However, the CT emission is the metastable excited state, and it requires special excitation energy to induce the corresponding emission, as shown in Figure 6. In the experiment, the broad excitation band peaking at around 366 nm (3.39 eV) provides a strong CT emission at around 507 nm (2.25 eV), which shows great agreement with our calculations. For the ^3^P_0,1_ emission of Bi^3+^ ions in CaZnOS, the dominant excitation and emission are both the ^1^S_0_ → ^3^P_0,1_ transition for Bi^3+^ substituting the Ca sites, as shown in Figure 6. Furthermore, our calculation shows that the emission energy of the CT excited state for Bi^3+^ in the Ca sites of CaZnOS:Bi^3+^ is similar to the ^3^P_0,1_ emission. The calculated energies of ^1^S_0_→^3^P_0,1_ excitation (3.23 eV) and CT emission (2.57 eV) are consistent with the measured optical transition bands peaking at 366 nm (3.39 eV) and 485 nm (2.56 eV). In the experiment, the CT excitation of Bi^3+^ in Ca sites was not obviously found in the excitation spectra, which may be covered by the stronger host-related excitation at around 275 nm (4.50 eV). It is noted that the dual-emission caused by the metastable excited state has also been observed in CaSnO_3_:Bi^3+^, while lower temperature is required to observe the emission with shorter wavelength [24].

**Figure 7:**
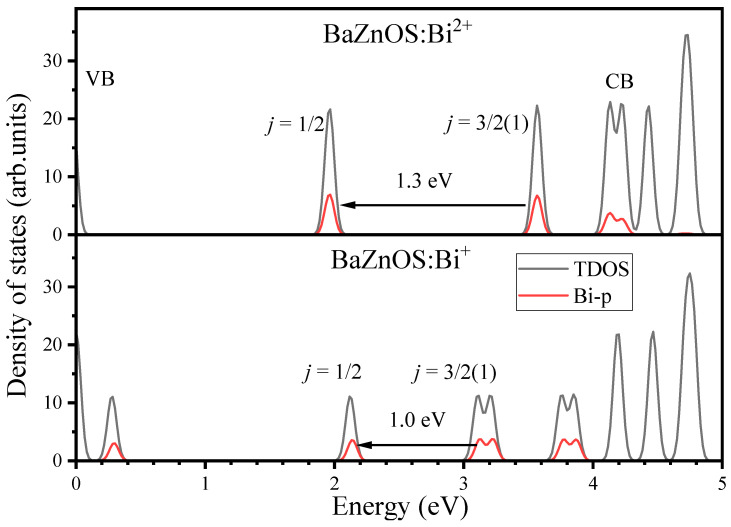
The emission of Bi^2+^ and Bi^+^ ions approximated by the splitting of the 6p Kohn–Sham orbitals.

Furthermore, since the lower valence-state Bi ions, such as Bi^2+^ and Bi^+^, were considered to be the reason for the extra red emission of BaZnOS:Bi, we studied their optical transition energies. The emissions of Bi^2+^ and Bi^+^ dopants in BaZnOS were estimated to be 1.6 eV and 1.0 eV, respectively, by the Kohn–Sham orbitals according to the methods of Ref. [27], as shown in Figure 7. Although the calculated ^2^P_3/2_(1) → ^2^P_1/2_ emission of Bi^2+^ was consistent with the measured emission band peaking at 627 nm, this does not support that emission origin from Bi^2+^ ions. First, the Bi^2+^ ions are not expected to be the dominant charge state in these two materials, as shown in the formation energy diagrams above. It requires a precise adjustment of the environment to meet the Fermi level for the formation of stable Bi^2+^ ions. Second, the measured excitation spectra show strong agreement with the Bi^3+^ ions, rather than that of the Bi^2+^ dopants in BaZnOS estimated by the Kohn–Sham orbitals. Third, the measured fluorescence decay curves [10] show the average decay time of emissions of 485 nm in CaZnOS:Bi (773 ns), 507 nm in BaZnOS:Bi (1.34 ns), and 627 nm in BaZnOS:Bi (884 ns). If the 627 nm emission is assigned to Bi^2+^ ions, the 485 nm emission in CaZnOS:Bi can also be assigned to Bi^2+^ ions considering the similar decay time. In short, with the combination of formation energy and optical transition energy calculations, we successfully reveal the site-selective occupancy and the luminescence processes, which helps to improve the reassignment of excitation and emission spectra and understand the dual-emission in the BaZnOS:Bi^3+^ system.

## 4. Conclusions

In our work, the first-principles calculations were performed to study the mono- and dual-emission properties of Bi^3+^ dopants in the oxysulfide *A*ZnOS (*A* = Ca, Ba). The local environments of the dopants and the formation energies of the defects in *A*ZnOS:Bi^3+^ systems were studied. The results show that the Bi^3+^ ions mainly occupy the Zn sites of BaZnOS hosts and the Ca sites of CaZnOS hosts. By calculating the defect levels and the electronic structures, the optical luminescence properties of Bi^3+^ dopants were obtained in the two hosts, which were in great agreement with the measurements. The excitation and emission bands were reassigned, where the ^3^P_0,1_ emissions were dominant in both CaZnOS:Bi^3+^ and BaZnOS:Bi^3+^ phosphors, acting as the emission band with lower energies. The additional emission band in BaZnOS:Bi^3+^ originates from the CT state, a metastable excited state. However, the CT emission energy of CaZnOS:Bi^3+^ is similar to that of ^3^P_0,1_ emission and the excited state is not as stable as BaZnOS:Bi^3+^. These all result in the difference between mono- and dual-emission in *A*ZnOS:Bi^3+^ (*A* = Ca, Ba) systems. Furthermore, the possibility of Bi ions with lower charge states, such as Bi^2+^ and Bi^+^, was discussed and excluded. Our results provide the basis for novel material design and emission tuning for Bi^3+^-doped materials.

## Figures and Tables

**Table 1:** The possible excitation and emission energies of Bi dopants in the two AZnOS (A = Ca, Ba) systems obtained with the PBE0+SOC functional.

		^3^P_0,1_	MMCT	CT
CaZnOS:Bi_Ca_	Excitation	3.23	/	3.95
	Emission	2.58	/	2.57
CaZnOS:Bi_Zn_	Excitation	2.31	4.29	3.54
	Emission	1.56	3.57	2.18
BaZnOS:Bi_Ba_	Excitation	/	/	3.68
	Emission	/	/	1.54
BaZnOS:Bi_Zn_	Excitation	2.29	3.67	3.60
	Emission	1.74	2.99	2.29

## Data Availability

The original contributions presented in the study are included in the article; further inquiries can be directed to the corresponding authors.
